# Biodiversity–function relationships in methanogenic communities

**DOI:** 10.1111/mec.14895

**Published:** 2018-11-22

**Authors:** Pawel Sierocinski, Florian Bayer, Gabriel Yvon‐Durocher, Melia Burdon, Tobias Großkopf, Mark Alston, David Swarbreck, Phil J. Hobbs, Orkun S. Soyer, Angus Buckling

**Affiliations:** ^1^ ESI and CEC, Biosciences University of Exeter Penryn UK; ^2^ School of Life Sciences University of Warwick Coventry UK; ^3^ Earlham Institute Norwich Research Park Norwich UK; ^4^ Anaerobic Analytics Ltd Okehampton UK

## Abstract

Methanogenic communities play a crucial role in carbon cycling and biotechnology (anaerobic digestion), but our understanding of how their diversity, or composition in general, determines the rate of methane production is very limited. Studies to date have been correlational because of the difficulty in cultivating their constituent species in pure culture. Here, we investigate the causal link between methanogenesis and diversity in laboratory anaerobic digesters by experimentally manipulating the diversity of cultures by dilution and subsequent equilibration of biomass. This process necessarily leads to the loss of the rarer species from communities. We find a positive relationship between methane production and the number of taxa, with little evidence of functional saturation, suggesting that rare species play an important role in methane‐producing communities. No correlations were found between the initial composition and methane production across natural communities, but a positive relationship between species richness and methane production emerged following ecological selection imposed by the laboratory conditions. Our data suggest methanogenic communities show little functional redundancy, and hence, any loss of diversity—both natural and resulting from changes in propagation conditions during anaerobic digestion—is likely to reduce methane production.

## 1 INTRODUCTION

Consistent with the large body of work on plant communities (Grime, 1997; Hector et al., 1999; Hooper, Adair, & Cardinale, 2012; Nielsen, Ayres, Wall, & Bardgett, 2011; Tilman, 1997), microbial diversity can have a positive role in a range of community functions, including aerobic respiration, litter decomposition and plant growth (Bell, Newman, Silverman, Turner, & Lilley, 2005; Delgado‐Baquerizo et al., 2016; Handa et al., 2014; Philippot et al., 2013; Wagg, Bender, Widmer, & van der Heijden, 2014). Strongly positive diversity–function relationships imply little functional redundancy of community members, and hence, loss of diversity resulting from environmental change may have considerable impact on community function (Jax, 2005; Loreau, 1998). One of the key microbial ecosystem functions where the role of diversity has not been experimentally investigated is methanogenesis: methane production resulting from the anaerobic conversion of H_2_, CO_2_ and short chain fatty acids by archaeal methanogens (Ferry, 2012). Methane is both a potent greenhouse gas and a renewable resource from organic waste; therefore, determining causal links between microbial community diversity, composition and methanogenesis is important.

Research investigating the links between methanogenesis and microbial diversity has been correlational. Studies of methanogenesis in natural soil communities have reported positive correlations between methane production (from an incubated soil sample) and the diversity of both methanogens and the total bacterial/archaeal communities (Wagner, Zona, Oechel, & Lipson, 2017; Yavitt, Yashiro, Cadillo‐Quiroz, & Zinder, 2012). However, any conclusions are potentially confounded by other environmental variables, such as pH, that can have a major role on community structure (Fierer et al., 2012; Hesse et al., 2018) and methanogenesis (Wagner et al., 2017). Other studies have focussed on correlations between community structure and methanogenesis under “common garden” laboratory conditions, where environmental factors are better controlled. The largest of these, involving 150 samples (Venkiteshwaran et al., 2017), showed no relationship between diversity and function, but in this case, the composition of communities differed in many ways in addition to diversity, and biomass was not controlled for.

As a consequence, there is a clear need to conduct manipulative experiments where causal links between diversity and methanogenesis can be determined. Manipulating diversity of methanogenic communities is nontrivial: They are typically very complex, consisting of varied taxa, most of which cannot be easily grown in pure culture or even cultivated at all. This makes the factorial manipulation of diversity at ecologically relevant levels almost impossible. Diversity can, however, be manipulated by dilution (Hernandez‐Raquet, Durand, Braun, Cravo‐Laureau, & Godon, 2013; Philippot et al., 2013; Salonius, 1981), which necessarily results in the loss of rare species relative to common species.

Here, we conduct such a dilution manipulation across six orders of magnitude on a methanogenic ancestral community obtained by mixing twelve separate communities. We have previously shown that mixing multiple communities maximizes the function and diversity in the mix (Sierocinski et al., 2017), thus using the mix maximized our chance of generating a highly functional community in the process. We allowed the biomass of the diluted cultures to become re‐established over months in laboratory reactors and then densities equalized between treatments. Methane production was subsequently measured over six weeks in laboratory anaerobic digesters. In an attempt to assess the importance of diversity of rare species relative to other differences in community composition, we also investigated correlations between diversity and methanogenesis in natural communities isolated from a range of industrial anaerobic digesters and associated feedstock environments (sewage, silage, slurry, etc) over eight weeks. A number of studies suggest that novel propagation conditions impose selection pressures can result in large changes in the composition of methanogenic communities (De Vrieze et al., 2015; Mladenovska, Dabrowski, & Ahring, 2003; Regueiro et al., 2012; Vanwonterghem et al., 2014), and hence, we determined community composition at the start and end of the experiment.

## 2 MATERIALS AND METHODS

### 2.1 Natural communities

In order to use samples that varied in diversity and methane production, we collected six pseudo‐pairs of anaerobic digester and feedstock samples. Anaerobic digester samples came from inside the fermentation tank, while feedstock samples were either acquired from the fermenter feedstock or fermenter seeding material (Table 1). The cultures were grown for eight weeks in 500‐mL bottles (total volume with headspace: 600 mL, Duran) using Automated Methane Potential Test System (AMPTS, Bioprocess Control Sweden AB) to measure CO_2_‐stripped biogas production. We confirmed that the resulting stripped gas was >95% methane by comparing the composition of the produced gas pre‐ and poststripping using GC‐FID (Agilent, 7890A) and comparing these with a standard curve made using methane standard (Sigma). Each sample was replicated in four fermenters, two of them fed using a synthetic medium displaying a C:N ratio of 15:1 and the other two with 30:1 C:N ratio. We used C:N ratios of 15 and 30 because they were reported to be close to optimal values for slurry (Hills, 1979; Hills & Roberts, 1981) and wastewater (Rughoonundun, Mohee, & Holtzapple, 2012)‐treating anaerobic digesters, respectively. Starting densities were equalized using 1xM9 salts (Na_2_HPO_4_•7H_2_O, 12.8 g L^−1^, KH_2_PO_4_ 15 g L^−1^, NaCl, 2.5 g L^−1^, NH_4_Cl, 5.0 g L^−1^) to 2 × 10^8^ [cells mL^−1^] based on qPCR measurements of 16S RNA genes (see below). 300 g of each sample was used as an inoculum and fed weekly with 25 mL of defined medium that mimicked the composition of standard anaerobic digester feeds composed of slurry and plant matter: meat extract 111.1 g L^−1^, cellulose 24.9 g L^−1^, starch 9.8 g L^−1^ glucose 0.89 g L^−1^, xylose 3.55 g L^−1^ for carbon‐to‐nitrogen ratio of 15:1 and meat extract 73.2 g L^−1^, cellulose 35.5 g L^−1^, starch 13.9 g L^−1^ glucose 1.27 g L^−1^, xylose 5.07 g L^−1^ for C:N ratio of 30:1 (all Sigma). Additionally, before the start of the fermentation, 0.3 mL of 1,000× trace metal stock (1 g L^−1^ FeCl_2_·4H_2_O, 0.5 g L^−1^ MnCl_2_·4H_2_O, 0.3 g L^−1^ CoCl_2_·4H_2_O, 0.2 g L^−1^ ZnCl_2_, 0.1 g L^−1^ NiSO_4·_6H_2_O, 0.05 g L^−1^ Na_2_MoO_4_·4H_2_O, 0.02 g L^−1^ H_3_BO_3_, 0.008 g L^−1^ Na_2_ WO_4_·2H_2_O, 0.006 g L^−1^ Na_2_SeO_3_·5H_2_O, 0.002 g L^−1^ CuCl_2_·2H_2_O) was added to each fermenter. The fermenters were run in two eight‐week batches.

**Table 1:** Description of anaerobic digester samples used in the experiment coupled with their paired natural samples

AD Sample ID	Location	Feed	Temp.	Time since last seeding [months]	Paired sample ID	Feedstock type
AD1	Farm	70% grass and maize silage; 30% food waste	42–44°C	14	AD7	Cow slurry
AD3	Farm	Maize; cow slurry; chicken manure	45°C	12	AD4	Maize; cow slurry; chicken manure
AD5	Sewage	Sewage sludge	36°C	12	AD14	Sewage sludge predigester
AD9	Sewage	Sewage slurry postdigester	36°C	60	AD8	Thickened sewage sludge
AD10	Farm	Food waste	36°C	18	AD11	Cow slurry
AD13	Farm	Maize/grass silage; cow slurry; chicken manure	40°C	5	AD12	Maize/grass silage; cow slurry; chicken manure

### 2.2 Dilution experiment

Initial inoculum was diluted by putting 3 ml into 100‐mL serum flasks with butyl rubber stoppers, containing 2.5 g of 15:1 C:N sugar mixture, 3 mL of 10x M9 salts (also see above), 21.47 g of sterile deionized water and 0.03 mL of 1,000× minerals solution (see above). The process has been serially repeated till the dilution of 10^−6^. Anaerobic conditions were ensured by filling the flasks with oxygen‐free nitrogen, and 1 mg/L resazurin was added to the medium to identify possible oxygen contamination. Six flasks of 10‐fold diluted culture were established and each independently serially diluted five times in ten‐fold steps by transferring 3 mL to produce dilutions ranging from 10^−1^ to 10^−6^. These dilutions have been incubated for three months at 35°C in order to regain the lost biomass. After that time, we measured the number of cells in each flask using optical density measurements at 600 nm (OD_600_) to make sure that they have regrown to measurable values, therefore showing that the diluted communities were still functional. Consequently, we transferred the cultures to AMPTS II system at equal densities. Cultures were fed weekly with the C:N 15:1 carbon source for 6 weeks, after which samples were isolated for the analysis of composition and cell density

### 2.3 DNA extractions

DNA was extracted using FastDNA^™^ SPIN Kit for Soil (MP) for the sequencing and Qiagen QiAamp DNA Stool Mini Kit for all the qPCR assays. The quality and quantity of the extractions were confirmed by 1% agarose gel electrophoresis and dsDNA BR (Qubit), respectively. We extracted DNA from one out of four replicates per community (a 15:1 C:N replicate) at the start and end of the experiment (pre‐ and postexperiment) involving the natural samples and from all samples at end of the experiment (postexperiment) in the dilution experiment.

### 2.4 Real‐time PCR assay

We used real‐time PCR followed by dilution to standardize starting microbial densities in the natural communities, because OD_600_ estimates of density would have been confounded by differences in the environments from which communities were sampled from. To ensure the method was accurate, we carried out a ten‐fold dilution series, confirmed by plating of a control bacterium, *Pseudomonas fluorescens* SBW25. We then extracted DNA of each dilution using the QiAamp DNA Stool Mini Kit. The DNA was amplified by qPCR using 16S rRNA primers 338f and 518r (Øvreås & Torsvik, 1998). The extracted dilution series (*Y* = −3.359 x log_(*X*)_ + 44.65; PCR efficiency = 98.5%; *r* ^2^ = 0.99) was compared to a curve of a DNA sample from slurry diluted after extraction (*Y* = −3.353*log_(*X*)_ + 13.52; PCR efficiency = 98.7%; *r* ^2^ = 1.0), indicating that the efficiency of the standard was comparable to the efficiency of the samples. PCR efficiency relates to the amplification per cycle efficiency, with 100% meaning doubling of DNA every cycle, the theoretical maximum.

For the postexperimental samples, we used a genomic DNA standard extracted from *P. fluorescens* for the Bacteria and *Halobacterium salinarum* for Archaea. The genome mass was calculated (Dolezel, Bartos, Voglmayr, & Greilhuber, 2003) and divided by the 16S gene copy number. DNA content (pg) = genome size (bp)/(0.978 × 10^9^) per copy number per gene of interest. The DNA quantity was measured using a Qubit dsDNA BR Assay Kit on a Qubit 2.0 Fluorometer, with the DNA diluted to concentrations containing 10^6^ 16S rRNA gene copies per μl and 10^7^ copies per μl for Archaea.

All pre‐experiment samples were run on a Stratagene MX3005P thermal cycler with 95°C for 3 min, followed by 40 cycles of 95°C for 15 s, 60°C for 20 s finalized by a melt curve of 95°C for 1 min and 55°C ramping up to 95°C (15 s for each step). All postexperiment samples were run on an Applied Biosystems StepOnePlus thermal cycler 95°C (3 min) 40 cycles 95°C (5 s), 60°C (10 s) flowed by a melting curve of 95°C (15 s) 60°C ramping up to 95°C in steps of 0.3°C (15 s for each step). The primers (Øvreås & Torsvik, 1998) used to identify Bacteria were 16S rRNA 338f—ACT CCT ACG GGA GGC AGC AG and 518r—ATT ACC GCG GCT GCT GG For Archaea, we used 931f—AGG AAT TGG CGG GGG AGC A and m1100r—BGG GTC TCG CTC GTT RCC. The following protocol was used: 1x Brilliant III Ultra‐Fast SYBR^®^ Green QPCR Master Mix, 150 nM 338f and 300 nM 518r or 300 nM 931f and 300 nM m1100r, ROX (30 nM for the MX3005P or 300 nM for the StepOnePlus), BSA 100 ng μL^−1^ final concentration. All samples were run in triplicates. The samples were compared to the standards using the software: MXPro MX3005P v4.10 Build 389 (Agilent) for the ancestral and stepone Software 2.3 for the descendant samples. Note slightly different methods were used because of a necessary change in equipment and that there were no direct comparisons between pre‐ and postexperiment samples.

### 2.5 Amplicon library construction and sequencing

16S rRNA gene libraries were constructed using primers designed to amplify the V4 region (Kozich, Westcott, Baxter, Highlander, & Schloss, 2013) (Table S1) and multiplexed. Amplicons were generated using a high‐fidelity polymerase (Kapa 2G Robust) and purified using the Agencourt AMPure XP PCR purification system and quantified using a fluorometer (Qubit, Life Technologies). The purified amplicons were then pooled in equimolar concentrations by hand based on Qubit quantification. The resulting amplicon library pool was diluted to 2 nM with sodium hydroxide and 5 μl transferred into 995 μl HT1 (Illumina) to give a final concentration of 10 pM. 600 μl of the diluted library pool was spiked with 10% PhiX Control v3 and placed on ice before loading into Illumina MiSeq cartridge following the manufacturer's instructions. The sequencing chemistry utilized was miseq reagent kit v2 (500 cycles) with run metrics of 250 cycles for each paired‐end read using miseq control Software 2.2.0 and RTA 1.17.28.

### 2.6 Analyses of sequenced samples

Overlapping 250‐bp paired‐end MiSeq amplicon reads were quality‐filtered and merged via the Illumina‐utils software (Eren et al., 2013) to generate high‐quality sequences spanning the V4 region. Quality filtering was only carried out on mismatches in the overlapping region between read pairs using a minimum overlap (–min‐overlap‐size) of 200 nt, a minimum quality Phred score (–min‐qual‐score) of Q20 and allowing a maximum of 5 mismatches per 100 nt (−*p* 0.05) over the 200‐nt overlapping region.

Read pairs passing the filtering criteria were merged and analysed using the Quantitative Insights Into Microbial Ecology (QIIME v.1.7) pipeline (Caporaso et al., 2010). Chimera checking and removal were done via the QIIME script *identify_chimeric_seqs.py* using the UCHIME reference “Gold” database. This step along with the OTU selection utilized USEARCH (Edgar, 2010; Edgar, Haas, Clemente, Quince, & Knight, 2011). OTU taxonomy assignment was performed via QIIME's *pick_open_reference_otus.py* function using the 13.8 version of the Greengenes database (McDonald et al., 2012), a 97% similarity threshold for OTU formation and a minimum cluster size of 2 (i.e., each OTU must contain at least two sequences). Technical replicates were collapsed, low abundance OTUs (<0.01% total) removed via *filter_otus_from_otu_table.py* (–min_count_fraction = 0.001) and samples rarefied to an even depth equivalent to the number of sequences present in the sample with the fewest number of reads (14,683 reads in total). The raw sequences are available online at the European Nucleotide Archive under Accession number ENA: PRJEB28621

### 2.7 Data analyses

Statistical analyses of community composition were performed in R (version 3.1.2; R Core Team, 2013) using the vegan (Oksanen, Kindt, & Legendre, 2007) and phyloseq (McMurdie & Holmes, 2013) packages. Following calculation of rarefaction curves in MacQIIME, a range of alpha diversity metrics were calculated: Simpson index (Simpson, 1949), OTU counts, Pielou evenness (Pielou, 1966) and phylogenetic diversity (Faith, 1992) were determined. Between‐community diversity was calculated using Bray–Curtis dissimilarity (Bray & Curtis, 1957), Jaccard Index (Jaccard, 1912) and UniFrac (weighted and unweighted), a phylogeny‐based dissimilarity matrix (Lozupone & Knight, 2005). The homogeneity of sample group dispersions (i.e., comparison of the magnitude of within‐community diversity) was determined using the vegan function *betadisper* and significance assessed using permutation tests (PERMDISP). Statistical significance of the sample groupings (i.e., ancestral–descendant communities) was determined via permutational multivariate analysis of variance (PERMANOVA) implemented in vegan as the *adonis* function (Oksanen et al., 2007). Mantel tests (Mantel, 1967) were used to assess the influence of community dissimilarity on difference in biogas production. For composition analysis, sequencing data were prefiltered to include only OTUs present at a frequency of more than 0.1% of total reads to avoid interferences from very rare OTUs, which may be errors. To determine whether there were any groups of organisms abundant in only one type of samples, communities were analysed at the phylum level using group_significance.py in MacQIIME. LefSE (Segata et al., 2011) was then used to determine differences in the frequency at the genus level between ancestral–descendant samples, as well as endpoint samples from good–bad gas producers (cumulative production of respectively more, or less than 3,000‐mL gas in the experiment). We also looked at the abundances of the archaeal reads, looking at the differences between the two types of methanogens: acetoclastic, that use acetate to produce methane, and hydrogenotrophic, that produce methane using carbon dioxide and hydrogen as substrate.

To determine how community composition affected gas production, cumulative gas production was independently regressed against diversity and density metrics. To determine functional saturation, the natural logarithm of cumulative gas production was regressed against the natural logarithm of species richness (Reich, Tilman, & Isbell, 2012). The value of the exponent (b) of this function is an indicator of the functional saturation.

We determined whether between‐community diversity was significantly different than null communities randomly generated from the data sets, after controlling for within‐community diversity (Chase, 2010). For the purpose of this comparison, we generated 1,000 null communities and used their mean as a community formed by pure stochastic process. We compared it with real‐life data, testing the null hypothesis that there is no difference between expected and observed between‐community diversity using the “oecosimu” function in vegan package for R (Oksanen et al., 2007).

In order to assess which factors are likely to be the main direct and indirect drivers of gas production in our correlational study, we applied path analysis (Grace et al., 2012; Yvon‐Durocher et al., 2015), where we use structural model equations using variables that had significant relationships with gas production (species richness, and archaeal and bacterial densities). We employed simplifying multiple hierarchical linear mixed effects models based on all combinations of plausible hypotheses (17, in total) about how the variables affect each other and gas production. Models were fitted using lme function in nmle package in R. We calculated the Akaike information criterion (AIC) scores of the models that were statistically significant using “lavaan” package for R (Rosseel, 2012) and used them to pick the model that best fitted the data. We compared the importance of particular paths in the final model using standardized coefficients that indicate a percentage change in gas production.

## 3 RESULTS

### 3.1 Natural communities

#### 3.1.1 Compositional changes through time

**Figure 1:**
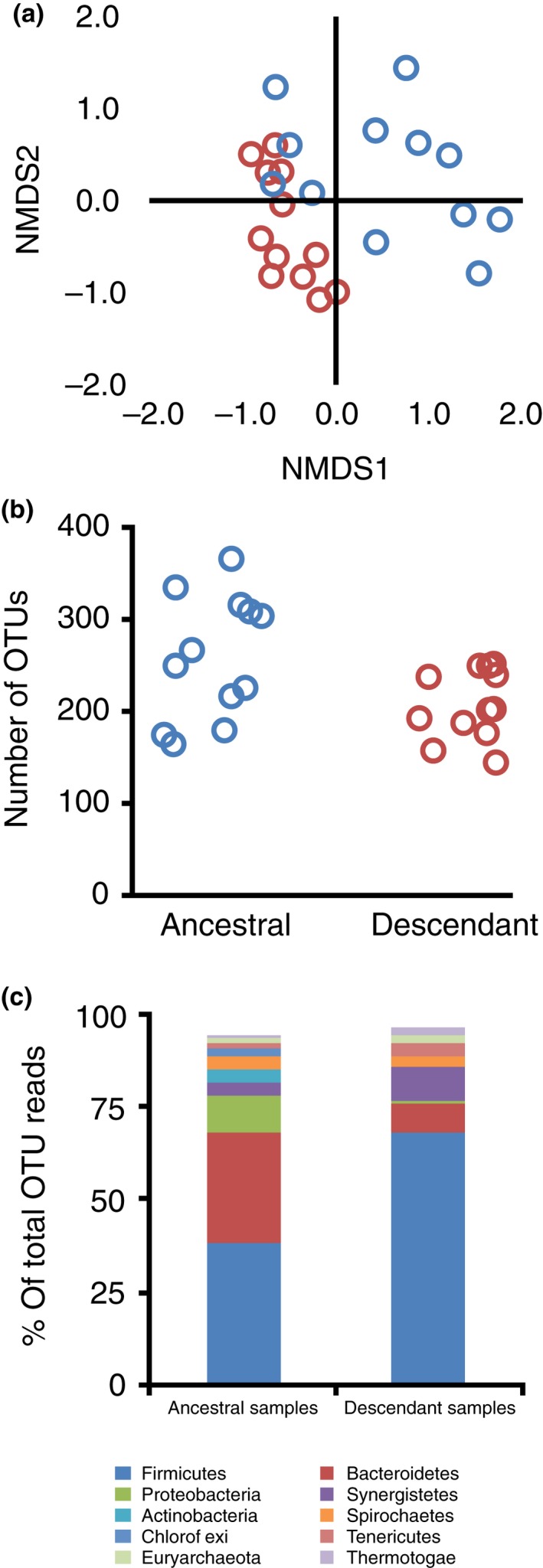
(a) Nonmetric multidimensional scaling (NMDS) plot of unweighted Bray–Curtis distances between ancestral (blue) and descendant (red) communities; (b) OTU number of ancestral (blue) and descendant (red) communities; (c) Mean frequency of the most common phyla in ancestral and descendant communities [Colour figure can be viewed at http://wileyonlinelibrary.com]

Community composition converged after eight weeks of cultivation as shown by the decrease in between‐community (beta) diversity of postexperiment communities comparing to pre‐experiment communities (Figure 1a; Permdisper, *F*_1,22 _= 12.38; *p *=* *0.002). There was also a moderate but significant separation between descendant and ancestral communities (Figure 1a; Bray–Curtis distance; PERMANOVA: *R* ^2 ^= 0.19, *p *<* *0.001). Note that this convergence was also robust to different distance measures: unweighted and weighted UniFrac and Jaccard index (*p *<* *0.01 in all cases). Net alpha diversity (OTU read counts and reciprocal Simpson's index) decreased between ancestral and descendant communities (*F*_1,11 _= 6.24, *p *=* *0.03; *F*_1,11 _= 6.97, *p *=* *0.02; Figure 1b), but community convergence was not simply the result of this loss of diversity, shown with a permutation test comparing observed beta diversity of descendant communities against null communities (*p *<* *0.01). The major change in community composition through time with respect to specific taxa was an increase in the frequency of *Firmicutes* reads (37.9%–68.0%), a decrease in the frequency of *Bacteriodetes* reads (30.1%–7.75%) and a decrease in the frequency of *Proteobacteria* reads (9.85%–0.86%) (Figure 1c). In general, we observed large changes in composition through time. A high proportion of OTUs were lost through time (between 34 and 72%). Similarly, a large proportion (between 17 and 65%) of OTUs that were present in the descendant samples were below detection levels in the ancestral samples. These changes had a significant impact on the community composition as shown by the difference between the pre‐ and postexperiment beta diversity when looking when using community distance is based on the presence/absence of taxa rather than relative frequency.

#### 3.1.2 Linking community composition with biogas production

We investigated how the characteristics of communities pre‐ and postexperiment affect total gas production. We used the cumulative gas production (averaged across replicates) value as the proxy for community performance for the tests, as there was little variability in terms of gas production ranks between weeks. For example, Spearman rank correlation coefficients between total, week one and week eight gas production ranged from 0.75 to 0.93, *p *<* *0.01 in all cases. Cumulative gas production was not affected by feed type (*F*_1,33 _= 1.33, *p *=* *0.3).

**Figure 2:**
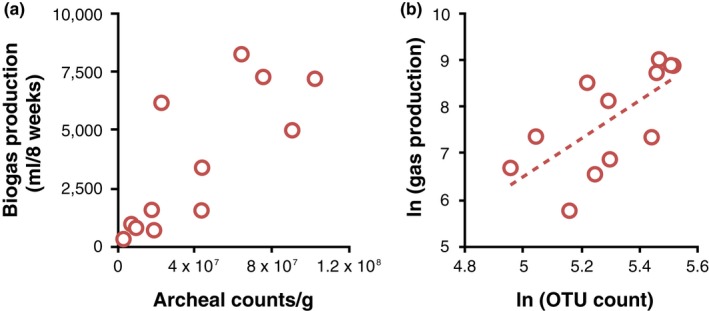
(a) Relationship between the counts of Archaea and biogas production; (b) Power curve of natural logarithms of gas production and richness of the descendant sample (OTU). b (slope) of the fitted linear trend = 4.07, *R* ^2 ^= 0.47, *F*_1,11_ = 8.8; *p *=* *0.0012 [Colour figure can be viewed at http://wileyonlinelibrary.com]

For starting communities, we found no significant correlations between gas production and either archaeal copy number, alpha diversity metrics (*p *>* *0.2 in all cases) or pairwise beta diversity (Mantel test: *r *=* *0.2; *p *=* *0.09). Note that bacterial copy number was equalized at the start of the experiment. By contrast, after eight weeks of propagation, there was a positive relationship between archaeal copy number and gas production (Figure 2a; *F*_1,10 _= 14.9, *p *=* *0.003; 83% of archaeal amplicon sequence reads were methanogens). There was no additional effect of bacterial copy number (*F*_1,9 _= 0.2, *p *>* *0.2). There were also no relationships between biogas production and alpha diversity metrics except for a positive relationship between gas production and species (OTU) richness (Figure 2b; *F*_1,10 _= 12.5, *p *<* *0.005). Unsurprisingly, communities that showed the greatest relative loss of OTUs through time produced the least gas (Spearman *R *=* *−0.67, *p *<* *0.05). The slope of the natural logarithms of gas production and OTU number was 4.07, suggesting an accelerating effect of increasing OTUs on methane production. Finally, pairwise beta diversity correlated with differences in gas production (Mantel *r *=* *0.54, *p *=* *0.001); the greater the difference in gas production, the bigger the difference in community composition.

We used path analysis to infer the likely causal relationships between gas production, species richness, and bacterial and archaeal densities. Comparisons of AIC scores (Table S3) of 17 hypothetical paths suggest that the interaction of bacterial biomass and species richness drives archaeal abundance, which leads to higher gas production (Figure S1).

#### 3.1.3 Linking biogas production to specific taxa

**Figure 3:**
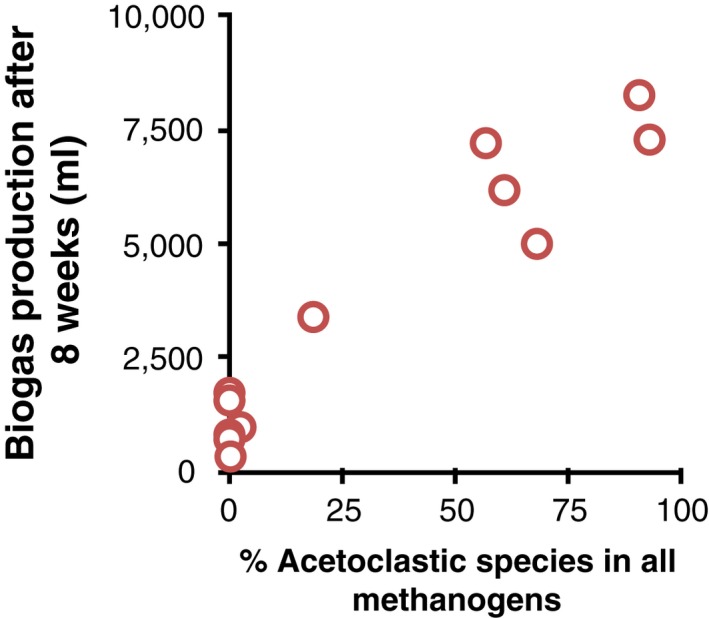
Relationship between the percentage of acetoclastic methanogen reads in total methanogen reads and gas production of a sample [Colour figure can be viewed at http://wileyonlinelibrary.com]

We also investigated how the frequencies of specific taxa might be associated with gas production (Table S2). Of particular note, there was a positive correlation between gas production and the proportion of *Methanosarcina,* a genus of acetoclastic methanogenic Archaea (*F*_1,10 _= 3.9, *p *<* *0.001; Figure 3).

### 3.2 Diluted communities

#### 3.2.1 Linking community composition with biogas production

**Figure 4:**
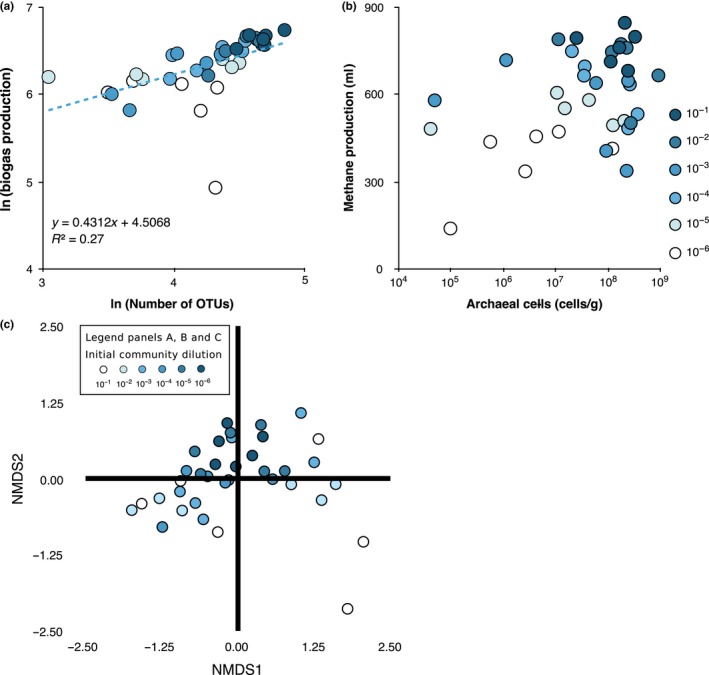
(a) Power curve of ln of cumulative biogas production [mL] and ln of the number of OTUs in the diluted communities. b exponent = 0.43, *R* ^2^ = 0.27, linear fit: *F*_1,35_ = 12.5, *p *<* *0.001. (b) Relationship between the biogas production and Archaea [cells/g sample]. Archaeal cell numbers plotted on log_10_ scale; (c) NMDS plots of Bray–Curtis based on the dilution treatment (see legend). Stress score is 0.14 [Colour figure can be viewed at http://wileyonlinelibrary.com]

To experimentally manipulate diversity, we diluted a mixed community over six orders of magnitude followed by a period of regrowth to allow equal numbers of cells to be inoculated across treatments. The manipulation worked: There were fewer OTUs detectable with increasing dilution (*F*_1,35 _= 21.8; *p *<* *0.001). Crucially, we found a positive relationship between biogas production and number of OTUs found in sample (*F*_1,35 _= 38.1; *p *<* *0.001). The slope of the natural logarithms of gas production and OTU number (*F*_1,35_ = 12.57, *p *=* *0.0012; *R* ^2 ^= 0.27; Figure 4a) was 0.43, suggesting an accelerating effect of increasing OTUs on methane production, suggesting little saturation of function with increasing OTU number. There were no significant relationships between biogas and other measures of diversity (*p *>* *0.1, in all cases) nor a relationship between biogas production and the number of bacteria present (*p *>* *0.1), as would be expected given that densities were equalized between dilution treatments. There was a positive relationship between biogas production and the number of total archaeal cells (*F*_1,35 _= 12.0; *p *=* *0.0015; Figure 4b), suggesting that increasing dilution reduced the equilibrium densities of archaeal cells. The dilution treatments show a small, but significant degree of separation (adonis, *R* ^2^ = 0.09, *p *=* *0.01, Figure 4c), but this was most likely a result of an increase in beta diversity with increasing dilution (Permdisp; *F*_5,30 _= 5.87; *p *<* *0.001), a limitation of multivariate ANOVA‐type analyses. These results held for other distance measures (*p *<* *0.001, in all cases).

#### 3.2.2 Linking biogas production to specific taxa

Three OTUs belonging to family *Coriobacteriaceae*,* Ruminococcaceae* and *Peptococcace*a were significantly overrepresented in the less diluted samples (Kruskal–Wallis test, Bonferroni *p *<* *0.05).

## 4 DISCUSSION

We investigated the link between microbial diversity and the rate of methane production in natural and manipulated communities. We found no correlations between any aspect of community composition at the start of the experiment and methane production across the 12 natural communities. However, after eight weeks of propagation in laboratory anaerobic digesters, there was a loss of diversity within communities and communities had converged. At this point, we found a positive relationship between methane production, species (OTU) richness, bacteria and methanogen density. We obtained the same qualitative results in communities where diversity was manipulated by dilution over six orders of magnitude. This suggests that decreasing species richness in methanogenic communities will reduce methane production and that this effect is robust to variation in species composition present in natural communities.

Manipulating diversity by dilution has limitations. Most obviously, it confounds diversity with species identity to some extent, in that dilution of communities results in the loss of predominantly rare taxa. As a consequence, the results suggest that methane production decreases with the increasing loss of rare species, rather than the loss of random taxa. To put this into context, the loss of half of the community made up by the rarest species results in approximately 50% reduction in gas production. Dilution also had the effect of increasing within‐treatment beta diversity, which could limit the interpretation of analyses. This increase in beta diversity is presumably the result of increased stochasticity in community assembly when taxa are at lower frequencies as a result of dilution.

The relationship between gas production and species richness in the dilution experiment showed little functional saturation (an exponent of 0.43 for the relationship) compared to most diversity–function studies (O'Connor et al., 2017). By contrast, the exponent of the gas production‐species richness relationship in the correlational study was extremely high (~4), suggesting an accelerating relationship. However, this very high value likely reflects an overestimation of species richness of the poorer‐performing communities. Specifically, poor‐performing communities had the greatest net loss of OTUs through time, and this loss may be underestimated because of residual DNA of dead cells and the presence of OTUs that were not yet driven to extinction. Our study supports the growing body of evidence that rare species play an important role in the community function (Lynch & Neufeld, 2015; Mouillot et al., 2013).

Both our studies that suggest large numbers of rarer species support higher densities of acetoclastic methanogens: methane‐producing Archaea locked into mutualisms with acetate‐producing bacteria (Ferry, 2012)), which are locked into syntrophic cross‐feeding interactions with acetate‐producing bacteria. Precisely why this might be is unclear, but recent theory suggests that growth under low energy conditions (as is the case under anaerobic conditions when oxygen is not used as the final electron receptor) is typically thermodynamically constrained, and results in a high diversity of metabolic niche specialists. This is because there a selective advantage to use a substrate in different way to competitors (negative frequency‐dependent selection (Clarke, 1979), to avoid thermodynamic inhibition of metabolism resulting from the build up of waste products (Großkopf & Soyer, 2016). More generally, thermodynamic constraints may help to explain why diversity seems less important for aerobic (Nielsen et al., 2011) than anaerobic (e.g., methanogenesis and denitrification; Philippot et al., 2013) functions in communities approaching natural levels of diversity. Finally, it is also possible that genetic variation within species, which would have been reduced by dilution and perhaps during propagation of the natural communities, could have contributed to the results. For example, recent work suggests that within‐species diversity associated with rapid adaptation can play a major role in the structure of natural soil microbial communities (Gómez et al., 2016).

The composition of the communities we investigated was broadly typical of methanogenic communities (Nelson, Morrison, & Yu, 2011; Yang et al., 2014; Yu, Lee, & Hwang, 2005), with *Firmicutes*,* Bacteroides* and *Proteobacteria* being the main phyla. However, consistent with other studies (De Vrieze et al., 2015; Demirel & Yenigün, 2006; Elbeshbishy, Nakhla, & Hafez, 2012; Mladenovska et al., 2003; Regueiro et al., 2012; Town, Links, Fonstad, & Dumonceaux, 2014; Vanwonterghem et al., 2014), we observed a convergence of communities through time. This was associated with an increase in *Firmicutes* and a decline in *Bacteriodetes* reads through time in the 12 natural communities. The most predominant group in the *Firmicutes*,* Clostridia, is* known for their cellulolytic and amylolytic activity (Nelson et al., 2011). Our medium was based on starch and cellulose, making *Clostridia* perfect candidates for the hydrolysis steps of fermentation within the system. Another reason for the increase in *Firmicutes* could simply be selection against them during sampling: *Firmicutes* have low oxygen tolerance (Kampmann et al., 2012), and while every care was taken during sampling*,* initial communities were inevitably exposed to air in the field. It is less clear why *Bacteriodetes* were selected against in the laboratory‐scale anaerobic digesters, but their reduction in frequency is consistent with increased biogas production: *Bacteroidetes* are associated with the production of propionate and other short fatty acids, which can lead to disturbances in anaerobic digester system (Gallert & Winter, 2008).

It was difficult to draw any firm conclusions about the role of specific taxa in gas production, beyond the positive effect of acetoclastic methanogens. However, in the natural converged communities, poor gas production was associated with the presence of *Pseudoramibacter*,* Oscillospira, Bacteroides uniformis* and *Enterobacteriaceae*. These species are typically associated with animal gut microbiomes, where they putatively are responsible for fermentation of glycans to butyrate (Benítez‐Páez, Gómez del Pulgar, & Sanz, 2017). It is possible that our medium, rich in meat extract, contributed to the enrichment of these species. The lack of animal host able to metabolize butyrate may have to its accumulation, detrimental to the functioning of the communities not capable of coping with it. OTUs that were overrepresented in the more diverse communities in the dilution experiment could plausibly have important roles: *Coriobacteriacea* have been suggested before to play a role in breaking down aromatic compounds in (Noguchi, Kurisu, Kasuga, & Furumai, 2014); *Ruminococcus* are involved in cellulolytic and xylolytic activity (Jia, Wilkins, Lu, Cai, & Lee, 2016); and *Peptococcus* are speculated to be acetate‐producing syntrophic partners of acetoclastic methanogens (Tang, Shigematsu, Morimura, & Kida, 2005).

The importance of rare species in determining the productivity of methanogenic communities has potentially important implications. First, communities may take a relatively long time to achieve maximal levels of methane production following environmental changes, given that key beneficial rare species may not be present. This is in contrast to aerobic communities where function is typically restored to high levels following environmental change because of functional redundancy within communities (Martiny et al., 2006; Strickland, Lauber, Fierer, & Bradford, 2009). Second, from a biotechnological perspective, we demonstrate, like research before us, that the starting inoculum plays a crucial role (De Vrieze et al., 2015; Elbeshbishy et al., 2012). Unfortunately, our results show that knowledge of the starting inoculum *a priori* may prove uninformative as the importance of community composition only becomes apparent after ecological selection imposed by the specific anaerobic digester conditions. This problem can be circumvented by inoculating multiple communities in the starting culture (Sierocinski et al., 2017). In summary, our results suggest that there is little functional redundancy in methanogenic communities, and hence, any loss of diversity will likely reduce methane production. Moreover, given that microbes appear to be dispersal‐limited to some extent (Bell, 2010), the potential for methanogenic communities to adapt to changing conditions is likely to be constrained by their starting composition.

## DATA ACCESSIBILITY

Sequencing data from these experiments will be stored in a publicly accessible repository. The raw sequences are available online at the European Nucleotide Archive under Accession number ENA: PRJEB28621.

## AUTHOR CONTRIBUTIONS

P.S., A.B., F.B., O.S.S., T.G. and P.J.H. conceived the experiments. P.S. and F.B. collected the data and conducted the experiments. M.B. performed the GC‐FID; M.A. and D.S. were responsible for sequencing and sequence data preparation. A.B., P.S. and G.Y.D. did the statistical analyses. A.B. and P.S. wrote the manuscript. All authors contributed to the revisions.
