# Supplementary material for: Biodiversity–function relationships in methanogenic communities
Source: Mol Ecol. 2018 Nov 22;27(22):4641–51. doi: 10.1111/mec.14895 (PMC6282539; doi:10.1111/mec.14895)
Supplement: Supplementary file 1 [file MEC-27-4641-s001.pdf]

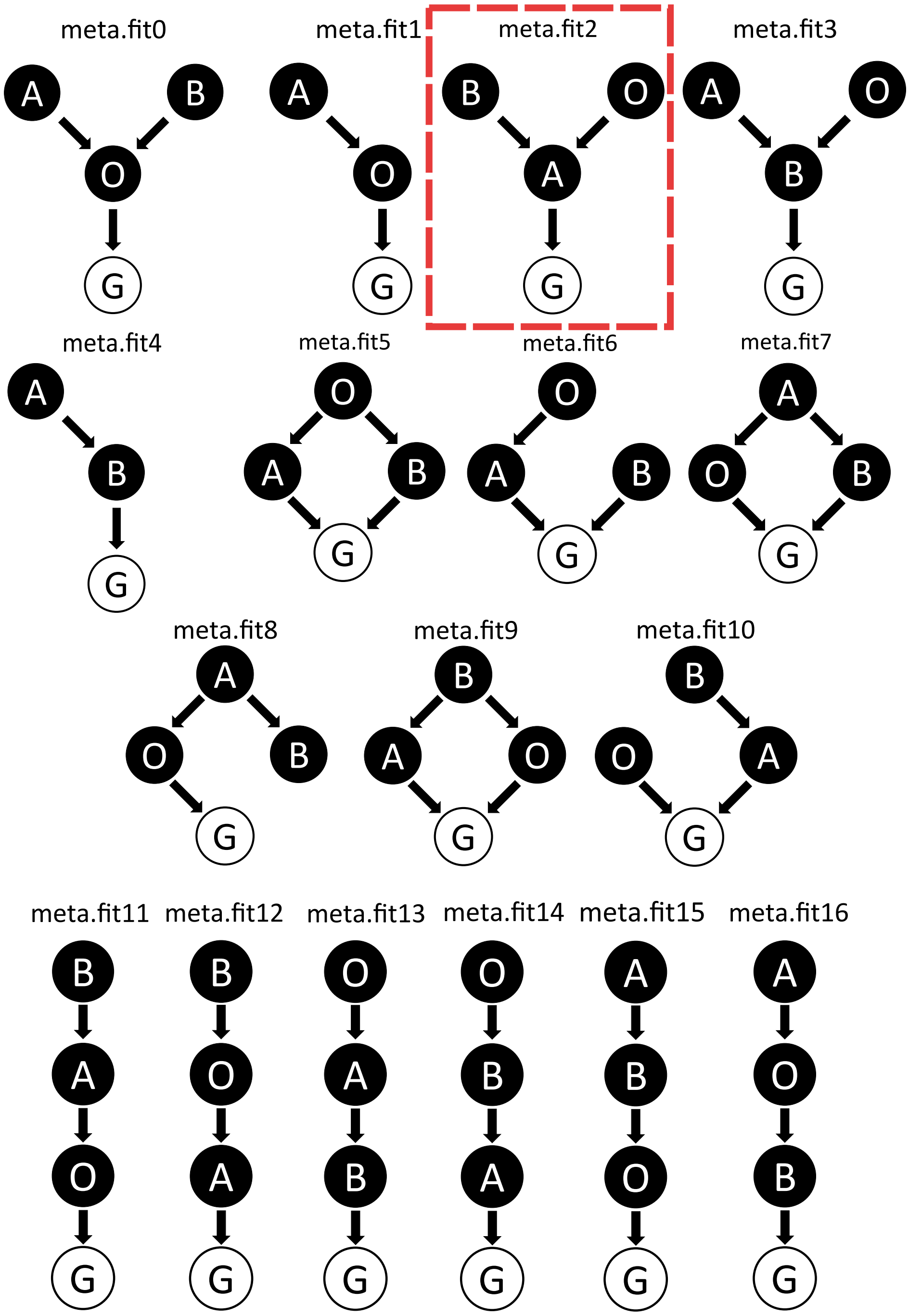

Figure S1: Interactions tested by the Path Analysis, best fitting model in red frame (see Table S3 for details).
A - $\log_{10}$ of Archaeal counts [cells/g]; B- Log $_{10}$ of Bacterial counts [cells/g];
O - Number of OTUs; G - Gas production [ml/8 weeks]

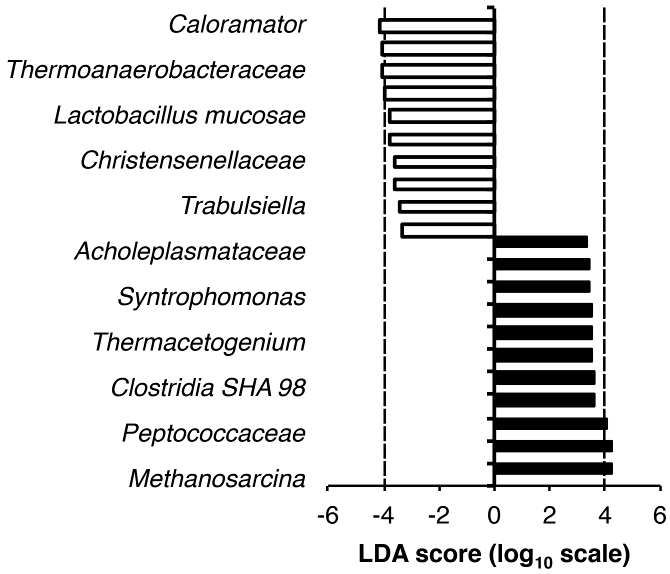

Figure S2: Overrepresented OTUs in high biogas samples (>3000 ml/8 week) - Black
in low biogas samples (<3000 ml/8 weeks) - White

Table S1: List of primers used

Primer name	Sequence
**Forward**	
v4.SA501	AATGATACGGCGACCACCGAGATCTACACATCGTACGTATGGTAATTGTGTGCCAGCMGCCGCGGTAA
v4.SA502	AATGATACGGCGACCACCGAGATCTACACACTATCTGTATGGTAATTGTGTGCCAGCMGCCGCGGTAA
v4.SA503	AATGATACGGCGACCACCGAGATCTACACTAGCGAGTTATGGTAATTGTGTGCCAGCMGCCGCGGTAA
v4.SA504	AATGATACGGCGACCACCGAGATCTACACCTGCGTGTTATGGTAATTGTGTGCCAGCMGCCGCGGTAA
v4.SA505	AATGATACGGCGACCACCGAGATCTACACTCATCGAGTATGGTAATTGTGTGCCAGCMGCCGCGGTAA
v4.SA506	AATGATACGGCGACCACCGAGATCTACACCGTGAGTGTATGGTAATTGTGTGCCAGCMGCCGCGGTAA
v4.SA507	AATGATACGGCGACCACCGAGATCTACACGGATATCTTATGGTAATTGTGTGCCAGCMGCCGCGGTAA
v4.SA508	AATGATACGGCGACCACCGAGATCTACACGACACCGTTATGGTAATTGTGTGCCAGCMGCCGCGGTAA
**Reverse**	
V4.SA701	CAAGCAGAAGACGGCATACGAGATAACTCTCGAGTCAGTCAGCCGGACTACHVGGGTWTCTAAT
V4.SA702	CAAGCAGAAGACGGCATACGAGATACTATGTCAGTCAGTCAGCCGGACTACHVGGGTWTCTAAT
V4.SA703	CAAGCAGAAGACGGCATACGAGATAGTAGCGTAGTCAGTCAGCCGGACTACHVGGGTWTCTAAT

V4.SA704	CAAGCAGAAGACGGCATACGAGATCAGTGAGTAGTCAGTCAGCCGGACTACHVG GGTWTCTAAT
V4.SA705	CAAGCAGAAGACGGCATACGAGATCGTACTCAAGTCAGTCAGCCGGACTACHVG GGTWTCTAAT
V4.SA706	CAAGCAGAAGACGGCATACGAGATCTACGCAGAGTCAGTCAGCCGGACTACHVG GGTWTCTAAT
V4.SA707	CAAGCAGAAGACGGCATACGAGATGGAGACTAAGTCAGTCAGCCGGACTACHV GGGTWTCTAAT
V4.SA708	CAAGCAGAAGACGGCATACGAGATGTCGCTCGAGTCAGTCAGCCGGACTACHVG GGTWTCTAAT
V4.SA709	CAAGCAGAAGACGGCATACGAGATGTCGTAGTAGTCAGTCAGCCGGACTACHVG GGTWTCTAAT
V4.SA710	CAAGCAGAAGACGGCATACGAGATTAGCAGACAGTCAGTCAGCCGGACTACHVG GGTWTCTAAT
V4.SA711	CAAGCAGAAGACGGCATACGAGATTCATAGACAGTCAGTCAGCCGGACTACHVG GGTWTCTAAT
V4.SA712	CAAGCAGAAGACGGCATACGAGATTCGCTATAAGTCAGTCAGCCGGACTACHVG GGTWTCTAAT

V4 Read 1 Seq Primer	TATGGTAATTGTGTGCCAGCMGCCGCGGTA*A
V4 Read 2 Seq Primer	AGTCAGTCAGCCGGACTACHVGGGTWTCTAA*T
V4 Index Seq primer	ATTAGAWACCCBDGTAGTCCGGCTGACTGAC*T

Table S3: AIC scores of the path analysis for each model

Model ID	K	AICc	Delta_AICc	AICcWt	Cum.Wt	LL
**meta.fit2**	7	-15.69	0	0.68	0.68	16.34
**meta.fit10**	7	-13.82	1.87	0.27	0.94	15.41
**meta.fit6**	7	-9.35	6.33	0.03	0.97	13.18
**meta.fit14**	9	-8.12	7.57	0.02	0.99	14.56
**meta.fit**	7	-6.04	9.65	0.01	0.99	11.52
**meta.fit9**	10	-5.1	10.59	0	1	15.41
**meta.fit3**	7	-2.84	12.85	0	1	9.92
**meta.fit12**	9	-2.19	13.5	0	1	11.6
**meta.fit11**	9	-1.83	13.86	0	1	11.42
**meta.fit5**	10	-0.64	15.05	0	1	13.18
**meta.fit7**	10	1	16.69	0	1	12.36
**meta.fit13**	9	1.37	17.06	0	1	9.82

**meta.fit15**	9	1.53	17.22	0	1	9.74
**meta.fit8**	10	1.7	17.39	0	1	12.01
**meta.fit1**	6	3.53	19.22	0	1	4.9
**meta.fit16**	9	10.66	26.35	0	1	5.17
**meta.fit4**	6	26.83	42.52	0	1	-6.75
